# A Lipid-Accumulating Alga Maintains Growth in Outdoor, Alkaliphilic Raceway Pond with Mixed Microbial Communities

**DOI:** 10.3389/fmicb.2015.01480

**Published:** 2016-01-07

**Authors:** Tisza A. S. Bell, Bharath Prithiviraj, Brad D. Wahlen, Matthew W. Fields, Brent M. Peyton

**Affiliations:** ^1^Department of Microbiology and Immunology, Montana State University, BozemanMT, USA; ^2^Energy Research Institute, Montana State University, BozemanMT, USA; ^3^Department of Civil, Environmental and Architectural Engineering, University of Colorado, BoulderCO, USA; ^4^Plant Biology Division, The Samuel Roberts Noble Foundation, ArdmoreOK, USA; ^5^Department of Chemistry and Biochemistry, Utah State University, LoganUT, USA; ^6^Center for Biofilm Engineering, Montana State University, BozemanMT, USA; ^7^Department of Chemical and Biological Engineering, Montana State University, BozemanMT, USA

**Keywords:** algal biomass, algal biofuel, bio-oil, bio-diesel, phycosphere

## Abstract

Algal biofuels and valuable co-products are being produced in both open and closed cultivation systems. Growing algae in open pond systems may be a more economical alternative, but this approach allows environmental microorganisms to colonize the pond and potentially infect or outcompete the algal “crop.” In this study, we monitored the microbial community of an outdoor, open raceway pond inoculated with a high lipid-producing alkaliphilic alga, *Chlorella vulgaris* BA050. The strain *C. vulgaris* BA050 was previously isolated from Soap Lake, Washington, a system characterized by a high pH (∼9.8). An outdoor raceway pond (200 L) was inoculated with *C. vulgaris* and monitored for 10 days and then the culture was transferred to a 2,000 L raceway pond and cultivated for an additional 6 days. Community DNA samples were collected over the 16-day period in conjunction with water chemistry analyses and cell counts. Universal primers for the SSU rRNA gene sequences for *Eukarya*, *Bacteria*, and *Archaea* were used for barcoded pyrosequence determination. The environmental parameters that most closely correlated with *C. vulgaris* abundance were pH and phosphate. Community analyses indicated that the pond system remained dominated by the *Chlorella* population (93% of eukaryotic sequences), but was also colonized by other microorganisms. Bacterial sequence diversity increased over time while archaeal sequence diversity declined over the same time period. Using SparCC co-occurrence network analysis, a positive correlation was observed between *C. vulgaris* and *Pseudomonas* sp. throughout the experiment, which may suggest a symbiotic relationship between the two organisms. The putative relationship coupled with high pH may have contributed to the success of *C. vulgaris*. The characterization of the microbial community dynamics of an alkaliphilic open pond system provides significant insight into open pond systems that could be used to control photoautotrophic biomass productivity in an open, non-sterile environment.

## Introduction

The cultivation of algal biomass has many industrial applications ranging from health-care products to biodiesel (Fields et al., 2014). As the number of applications and subsequent demand for biomass increases, a challenge will be to exponentially increase production cost-effectively. The use of alkaliphilic photoautotrophs may help overcome some of the constraints associated with large-scale biomass production in open systems due to limited niche accessibility caused by higher pH values. Algal production of triacylglycerol (TAG) and other lipids are of substantial interest because of being biodiesel precursors that can be transesterified into fatty acid methyl esters (FAMEs; Sheehan et al., 1998; Dismukes et al., 2008; Scott et al., 2010). Many studies have screened algal species for biodiesel applications based on high lipid content (Sheehan et al., 1998; Dismukes et al., 2008; Griffiths and Harrison, 2009; Scott et al., 2010; Griffiths et al., 2011), and *Chlorella vulgaris* has been identified as one such species (Li et al., 2008; Griffiths et al., 2011; Duong et al., 2012).

Many studies on *C. vulgaris* and other species have been conducted in closed photobioreactors (PBRs) whose overall costs are considered a major constraint in the scale-up of algal biodiesel production (Smith et al., 2010; Kazamia et al., 2012a; Chisti, 2013; Shurin et al., 2013). However, in an effort to scale up production at lower capital investments, open pond systems have been shown to be a viable alternative (Smith et al., 2010). These open systems are, however, prone to colonization by environmental microbes spanning all three domains, and may contain hundreds of distinct taxa whose relative abundances vary by orders of magnitude (Fulbright et al., 2014). By employing parameters from “extreme” environments observed in natural systems, such as high pH, unwanted colonization (e.g., invasion) may be limited (Georgianna and Mayfield, 2012; Wang et al., 2013; Selvaratnam et al., 2014). In addition, alkaline systems favor higher dissolved inorganic carbon (DIC) from atmospheric CO_2_ thereby providing increased carbon delivery for primary producers. Moreover, some of the highest primary production rates have been reported for microbial, alkaline systems (Melack and Kilham, 1974; Hem, 1985).

Bacterial colonization has benefits and drawbacks for biomass production that are determined by the system’s community structure and composition. Numerous studies have documented positive, symbiotic relationships between algal taxa and bacteria (Croft et al., 2005; Watanabe et al., 2005; Sapp et al., 2007; Xie et al., 2013). Specifically, different species of *Pseudomonas* have been observed living in association with algae, including *C. vulgaris* (Sapp et al., 2007). The physical association, in which the bacteria live in the exopolymeric substances (EPS) or “phycosphere” of the alga, has been shown to increase *C. vulgaris* growth (Guo and Tong, 2013). The alga not only benefits from exchange of growth promoting and antibacterial metabolites in the niche space of the phycosphere, but also via the exclusion of potential opportunistic pathogens (Kazamia et al., 2012a,b; Smith and Crews, 2013). Additional work has shown symbiosis to be critical for adaptation to thermal stress resulting in higher algal biomass (Xie et al., 2013). These mutual positive relationships may have benefits for biotechnological applications offering the potential to artificially select microbial consortia that promote the growth of desired species (Kazamia et al., 2012a, 2014; Ortiz-Marquez et al., 2012; Natrah et al., 2013; Santos and Reis, 2014).

In the described study, we utilized pyrosequencing to monitor fluctuations in the community structure of an outdoor raceway pond inoculated with *C. vulgaris* during scale-up from 200 to 2,000 L. The results indicated that the inoculated algal population could maintain predominance under alkaline conditions, and that bacterial diversity increased while archaeal diversity decreased over time. In addition, particular populations could be correlated with *C. vulgaris*.

## Materials and Methods

### Site Description and Raceway Pond Conditions

Outdoor ponds were located in Logan, Utah (July 2011) approximately 40 km west of the northern arm of the Great Salt Lake (GSL). The 200 L oblong pond manufactured by Separations Engineering Inc. was lined with fiberglass and equipped with a paddle wheel promoting gas exchange with ambient air (Separatons Engineering Inc., San Diego, CA, USA). Initially, a 200 L raceway was inoculated with *C. vulgaris* (10% v/v), maintained at a culture depth of 13 cm, and monitored for 10 days. On day 10, the entire culture was transferred into an adjacent 2,000 L raceway and maintained at a culture depth of 20 cm for the remaining 6 days of the experiment. The 2,000 L raceways were constructed of cinder blocks stacked two high with 46 mil EPDM rubber pond liner creating the pond. Marine board was used to divide the pond into a circulating raceway with a paddlewheel providing circulation. The ponds were inoculated with *Chlorella vulgaris* BA050 that was previously isolated from Soap Lake, Washington, which is characterized by growth at high pH (∼9.8) (Dimitriu et al., 2008). The isolate was maintained on agar plates and was streak isolated during each subsequent plating every 2 months. The 18S rRNA gene sequences obtained from isolated DNA confirmed the presence of a single eukaryotic microorganism. A single 200 L raceway was inoculated with a 20 L culture (10% volume) that had been previously cultivated in shaker flasks bubbled with 1% CO_2_. A more saline version of Bold’s Basal Medium, consistent with the salinity of seawater (35 ppt), at pH 8.7 was prepared under non-sterile conditions with the addition of dry salts and concentrated solutions (Nichols and Bold, 1965). Inoculation resulted in a cell density of 3.6E+6 cells/mL with the addition of sufficient medium to bring the total volume to 200 L. The pH was not controlled in the pond. Unfiltered tap water was added each day to replace measured evaporative loss. After 10 days, repeating methods from the initial inoculation, the 2,000 L pond was inoculated by transferring 200 L (10%) of culture from the first pond (200 L).

### Sample Collection

Samples were collected twice daily for cell density ascertained by OD_750_ and direct cell counts via optical microscopy (cells/mL). Additionally, 500 mL samples were collected and frozen at -80°C for DNA extraction and 454 sequencing.

### DNA Extraction and Sequencing

#### DNA Extraction

Samples were slowly thawed at 4°C and microbial biomass was collected via filtration through 0.22 μm polyethersulfone membrane filters. The solids were then suspended in the MO BIO PowerMax^TM^ Soil DNA Isolation Kit PowerBead Solution, and the cells were lysed via three cycles of liquid nitrogen freeze–thaw and ground with a mortar and pestle aided by sterile sand (Zhou et al., 1996) (MO BIO Laboratories Inc., Carlsbad, CA, USA). The DNA was cleaned and concentrated with the Wizard^®^ SV Gel and PCR Clean-Up System (Promega Corporation, Madison, WI, USA) according to the manufacturer’s protocol.

#### Bar-Coded Pyrosequencing

Pyrosequencing was utilized to characterize the microbial population of the ponds. PCR was used to increase the DNA concentration needed for pyrosequencing analysis. Each sample was labeled with a unique 10 nucleotide-barcode for multiplexing. The SSU rRNA gene sequences for *Eukarya* and *Bacteria* were amplified via 25 cycles of PCR with the following barcoded primers; 7F (5′-ACCTGGTTGATCCTGCCAG-3′) and 591R (5′-GGAGCTGGAATTACCG-3′) for *Eukarya* and FD1 (5′-AGAGTTTGATCCTGGCTCAG-3′) and 529R (5′-CGCGGCTGCTGGCAC-3′), which targeted the V1–V3 region of *Bacteria* (Bowen De León et al., 2012). Archaeal sequences were amplified separately from *Bacteria* using a nested approach with non-barcoded 21F (5′-TTCYGGTTGATCCYGCCRGA-3′) and 1492R (5′-CGGTT ACCTTGTTACGACTT-3′) for 20 cycles followed by an additional 20 cycles with barcoded 751F (5′-CCGACGGTGAGRGRYGAA-3′) and 1204R (5′-TTMGGGGCATRCNKACCT-3′) (Baker et al., 2003; Barnhart et al., 2013). PCR products of the correct size were confirmed using a 1% agarose gel. Products were cut from the gel and pooled using an Ultrafree-DNA gel extraction column (Millipore Corporation, Bedford, MA, USA). The gel extract was cleaned and concentrated using the Wizard^®^ SV Gel and PCR Clean-Up System, and dsDNA was quantified with a Qubit fluorometer (Invitrogen, Carlsbad, CA, USA). Adaptors for 454 sequencing were ligated to the amplicons and were pyrosequenced on a 454 GS-Junior (454 Life Sciences, Branford, CT, USA). Roche’s image analysis separated sequences by barcode. Sequences were trimmed to one standard deviation below the mean length or removed if shorter. Employing the Phred score filter, 15% of the nucleotides were allowed to be below Q27, and removed if primer errors or Ns were observed.

### Bioinformatic Sequence and Community Analysis

Data analysis was performed using the Quantitative Insights into Microbial Ecology (QIIME) software package, version 1.4.0 (Caporaso et al., 2010b). Parameter settings for demultiplexing were at a default level of 200 and 1000 bp in length. Metadata files were prepared according to a QIIME compatible template taking into account environmental sampling data on pH, temperature, and ionic concentrations. Libraries were split according to barcode for each of the respective domains (*Archaea*, *Bacteria*, and *Eukarya*). Sequences were then concatenated for data normalization needed in downstream analysis. Operational taxonomic units (OTUs) were assigned using the closed reference OTU picking protocol. Clusters were referenced against the Silva 108 database and pre-clustered at 97% identity using UCLUST (Edgar, 2010).

Sequence reads that matched a Silva reference sequence at 97% identity were clustered within an OTU defined by a reference sequence. OTU assignment (and all subsequent steps) was performed for the combined *Archaea*, *Bacteria* and *Eukarya* reads. The singleton OTUs were discarded. The centroid sequence in every cluster was selected to represent the cluster and aligned with the Silva core set using PyNAST (Caporaso et al., 2010a). Chimeric sequences, identified with Chimera Slayer (Haas et al., 2011) and reads that failed to align with PyNAST were excluded from subsequent analyses. PyNAST (v1.1) was used for sequence alignment and filtering through QIIME using default parameters.

Taxonomic assignments were additionally assigned using the retrained RDP Classifier (Wang et al., 2007) on the Silva 108 database for phylogenetic resolution at the genus level. Taxonomic summary for *Archaea*, *Bacteria*, and *Eukarya* was plotted to the genus level with a given relative abundance based on diversity and distribution pattern per domain. Taxa distribution was also summarized by time (sample day). The biodiversity analysis downstream between samples was derived using UniFrac (Lozupone and Knight, 2005) that took into account the phylogenetic structure of the algal pond microbial communities. Taxonomic richness was calculated by a rarefaction analysis based upon OTU tables that were rarefied at an even sampling threshold value. Richness was measured on the basis of the Chao index (Chao et al., 2010).

The co-occurrence of community members was illustrated in a heat-map using the R vegan package version 2.0-10 (Oksanen, 2011). Due to the fact that some taxa had a 0% relative abundance at certain time points, 0.1 was added to all values in order to be log transformed. In an effort to enhance the visual distribution of taxa, log transformed values were cubed and resulting values plotted (log(relative abundance + 0.1)^3^). A SparCC analysis was subsequently used to construct community correlation networks by estimating linear correlation values between log transformed abundances based on the absolute number of sequences for an OTU rather than a relative abundance (Friedman and Alm, 2012; Berry and Widder, 2014). The key advantage of this analysis was that, for instance, the ratio of the fractions of two OTUs was independent of the fluctuations in other OTUs included in the analysis (i.e., subcompositional coherence) (Friedman and Alm, 2012). Archaeal taxa were not included in this analysis due to the sharp decline in relative abundance between days 1 and 3 and near absence by the end of the pond experiment. We observed that the drastic decline in Archaeal relative abundance would have produced deceptive relationships in the network model.

### Statistical Analysis

A principal coordinate analysis (PCA) was used to reduce dimensionality and give structure to the water chemistry variables obtained from each time point (Legendre and Gallagher, 2001). In order to incorporate taxonomic data, we used the direct-gradient ordination technique, Canonical Correspondence Analysis (CCA), which concurrently showed pond taxa, time points, and water chemistry (Hall and Smol, 1992). This kind of ordination is appropriate when assessing community dynamics because it does not use Euclidean based metrics that assume linear trends in community change. Not only does the ordination show environmental factors influencing community change, but results suggest potential interactions between taxa (Amaral-Zettler et al., 2010). The first two axes, CCA1 and CCA2, typically account for the majority of observed variation. All axes are constrained to present a linear combination of the water chemistry that maximizes the dispersion of taxa (Hall and Smol, 1992).

## Results and Discussion

### Environmental Variables

Water chemistry was monitored daily over the course of the experiment and fluctuations were used to draw correlations to community structure (**Table 1**).

**Table 1:** Water chemistry over the course of the pond run.

(mg/L)	Day 1 (200 L)	Day 3 (200 L)	Day 7 (200 L)	Day 8 (200 L)	Day 11 (2,000 L)	Day 16 (2,000 L)
**F-**	0.05	42.9	71.6	0.6	71.6	225.7
**Cl-**	8092	1405	1294	2460	1114	537
**NO_3_^-^**	13.02	60.14	40.98	8.68	210.24	191.50
**SO_4_^-^**	427.20	277.44	275.52	345.60	300.48	335.04
**PO_4_^-^**	10.56	0.96	b.d.	0.29	9.60	6.72
**P**	5.92	2.85	1.46	1.14	5.26	2.90
**Ca**	52.4	22.1	14.8	12.4	41.6	20.3
**K**	198	226	262	213	302	382
**Mg**	93.1	116.0	110.0	97.9	144.0	178.0
**Na**	4734	5316	5527	4740	6211	7483
**S**	157	150	148	135	175	219
**pH**	8.24	9.77	10.41	10.69	8.39	10.02


*Values are in mg/L. b.d. stands for below detection. Volume of pond indicated by 200 or 2,000 L.*

The nitrate concentration was lowest on day 8 as *C. vulgaris* achieved stationary phase in the 200 L raceway. Changes in fluoride and chloride anion concentrations at this time may have been due to the use of tap water to compensate for evaporative loss. Nitrogen concentrations recover on day 10 when the culture is transferred to the 2,000 L raceway and combined with new media. The pH values remained high over time and may have benefited *C. vulgaris* (*R*^2^ = 0.2). The decline in pH from 10.7 to 8.39 on day 11 corresponded with the transfer of the pond from the 200 to 2,000 L raceway.

### Community Composition and Interaction

The SSU rRNA for *Bacteria*, *Archaea*, and *Eukarya* were amplified and sequenced for each of the six sample days. After screening sequences for errors (see Materials and Methods), 161,731 quality gene sequences with a valid barcode were retrieved. Sequences with a 97% identity were clustered within an OTU totaling 1,349 observed OTUs composed of 748, 249, and 352 OTUs of *Bacteria*, *Archaea*, and *Eukarya* respectively for all sampled days (**Table 2**).

**Table 2:** 454 pyrosequencing statistics.

Number of samples	18
Number of OTUs	2,074
Number of total sequences	161,731
Minimum sequences/sample	303
Maximum sequences/sample	22,256
Sequences/sample	8,985
Standard deviation	5,626
Even sampling depth	3,068


**FIGURE 1:**
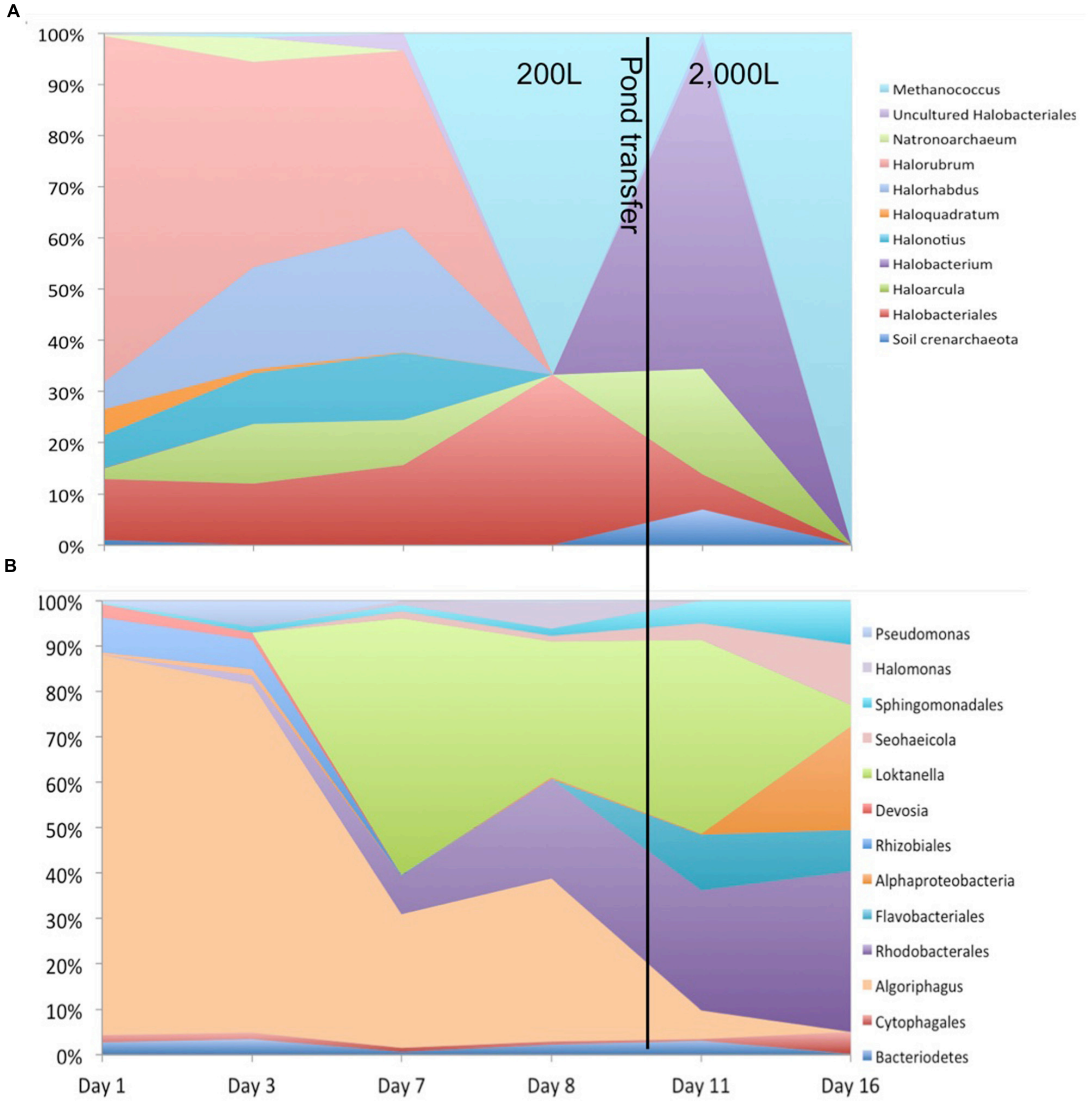
**Total relative abundances of **(A)** archaeal and **(B)** bacterial taxa by order (as order, genus, or unknown) with >2% relative abundance over the 16 day sample period**.

**Figure 1** shows the relative abundances for the observed archaeal and bacterial taxa.

**FIGURE 2:**
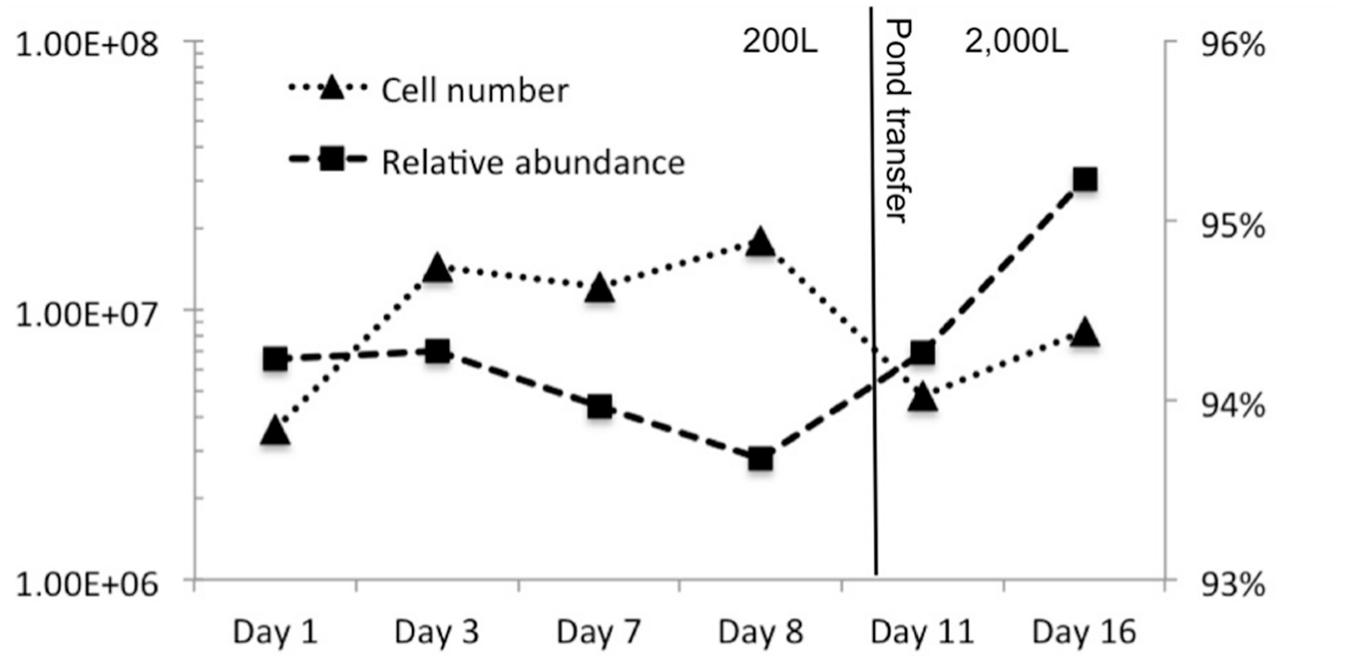
**Cell concentration (log cells/mL) and the relative abundance of *C. vulgaris* over 16-day sample period.** The transfer from the 200 to 2,000 L raceway is indicated by the vertical line.

**Figure 2** shows phosphate limitation in the plateau of *C. vulgaris* observed from day 3–8, emulating nutrient conditions frequently observed in wastewater and some natural systems. Nitrogen availability has more than doubled due to anthropogenic inputs facilitated by the Haber–Bosch process entering aquatic systems through precipitation, runoff, and dust deposition (Baron et al., 2000; Wolfe et al., 2001; Fenn et al., 2003; Gardner et al., 2008; Miller and McKnight, 2012; Brahney et al., 2015). Thus, phytoplankton is typically phosphorus limited giving a competitive advantage to taxa that can quickly scavenge phosphorus (Baron et al., 2000; Wolfe et al., 2001; Elser et al., 2009). In addition, some *C. vulgaris* strains have been shown to accumulate polyphosphate (Aitchison and Butt, 1973). Considering that nitrogen inputs are nearly unavoidable in the majority of open systems, most communities will be phosphorus limited, the effects of which are pertinent to endeavors such as commercial algae production in open systems. Moreover, photoautotrophs that have the ability to accumulate and/or scavenge phosphorus will be more competitive in open, mixed systems in which low phosphate levels can be used. Combined, these attributes reduce the need for higher levels of phosphorus and/or the use of low-quality phosphorus.

Effects of phosphorus limitation on *C. vulgaris* did not appear to detrimentally affect ability to compete and grow under the tested conditions as it was observed to be the single dominant Eukaryote during the 16-day experiment. **Figure 2** shows that *C. vulgaris* recovered from phosphorus limitation upon transfer to the 2,000 L pond, and throughout the pond experiment it was the dominant eukaryotic taxon and did not decline below 93% of eukaryotic relative abundance. The other 7% was composed of pine pollen, insects, and fungi. While it is difficult to ascertain physiology from phylogeny, sequences indicative of *Psuedomonas* were inversely correlated to high phosphate levels (Istvánovics, 2008). Organisms from this genus can be phosphate-accumulating and/or grow under low-phosphate conditions (Sidat et al., 1999). It is likely that phosphate-accumulating bacterial populations would be selected as overall P levels are depleted. As expectations for inexpensive biomass and feedstock become greater, we will need improved insight into biological responses to low-level and low-quality phosphorus.

**FIGURE 3:**
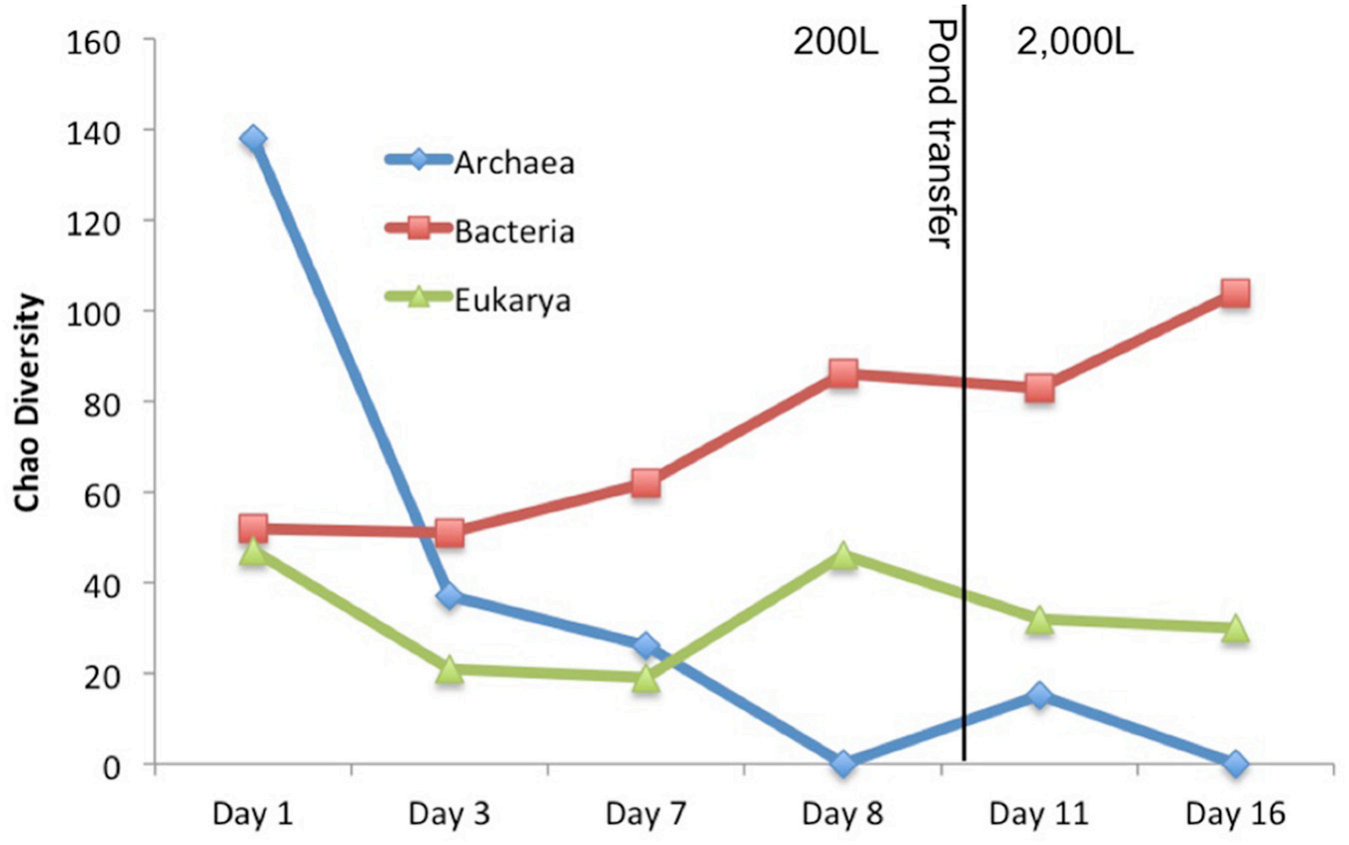
**Chao diversity for each domain plotted over time.** Archaea started with high diversity but quickly declined. Eukarya maintained a steady diversity level composed almost entirely of *C. vulgaris*. Bacterial diversity steadily increased with time.

Other organisms propagated in the pond but did not appear to have a detrimental effect on the alga population. Several halophilic archaeal taxa were observed in the first three time points, especially day 1 leading to the greatest observed diversity that declined over time (**Figure 3**). In addition to the Archaea, several bacteria were present in the first sample. The high archaeal and bacterial diversity did not appear to have a negative effect on *C. vulgaris*, which was the predominant eukaryote, and minimal fluctuation was observed in the eukaryotic diversity. As the Archaeal taxa diversity declined, the bacterial diversity increased (**Figure 3**). It is not known if the decline in archaeal populations was related to the increase in bacterial populations or an independent process, such as lower salinity.

The transitory presence of halophilic taxa could have been due to suboptimal conditions in the ponds. These halophilic microorganisms have an optimal pH range from 6.8 to 9, NaCl concentration between 3.4 and 5.1 M, and generally require at least 0.85 to 3.4 M in order to maintain osmotic pressure for cell integrity and prevent lysis (DasSarma and DasSarma, 2006; Oren, 2006; Bowers and Wiegel, 2011; Cui et al., 2012). Their presence in the ponds could potentially be due to the proximity of the pond to the GSL that is 40 km to the west and characterized by high salinity and a neutral pH (Post, 1977; Oren, 1994, 2006; Baxter et al., 2005; Tazi et al., 2014). Wind dispersal and precipitation events may have been responsible for the presence of these populations in the pond. We observed numerous haloarchaeal genera including *Halorubellus*, *Haloquadrata, Halalkalicoccus, Candidatus, Halomonas, Halobacterium*, and *Haloarcula* and the halobacterial genera *Devosia, Aliihoeflea, Halomonas sp., Seohicola, Eruthromicrobium, Aquiflexum*, and *Rhodobacterales* which have also been found in GSL samples (Post, 1977; Oren, 1994, 2006; Baxter et al., 2005; Tazi et al., 2014). However, another possibility is that these taxa were already present in the salts used to make the medium. The vast majority of these taxa subsisted for only the first two time points (**Figure 3**). It is unknown if the detected sequences were indicative of populations that survived for a given time in the test pond or simply were static and/or dead cells that were transported to the ponds.

In contrast to the halophilic microorganisms, *C. vulgaris* has been shown to be inhibited by concentrations greater than 1 M NaCl and showed substantial declines in cell concentration at 0.5 M (Alyabyev et al., 2007). The highest recorded salinity in the ponds was 0.7 M with an average 0.2 M. Thus, the success of *C. vulgaris* in the ponds further supports that salinity was below the presumptive optima of the halophiles. The changes in archaeal and bacterial community structure did not appear detrimental to *C. vulgaris* and a high relative abundance (>93%) was observed throughout the course of the pond experiment. A decrease in cell number was observed immediately after pond transfer and was likely due to dilution of cells during the transfer of the 200 L inoculum to the 2,000 L pond; however the relative abundance of *C. vulgaris* remained consistent (94–95% relative abundance).

### Correlation of Environmental Variables and Community Structure

Environmental factors could have also played a role in the success of *C. vulgaris.* We observed high pH values which ranged between 8.2 and 10.7 (**Table 1**), which may have prevented other algal taxa from successfully colonizing the pond. This finding demonstrates that the cultivation of a single algal strain in an open alkaline pond without the addition of antibiotics or herbicides can be successful (Lundquist et al., 2010; Smith et al., 2010; Smith and Crews, 2013; McBride et al., 2014).

**FIGURE 4:**
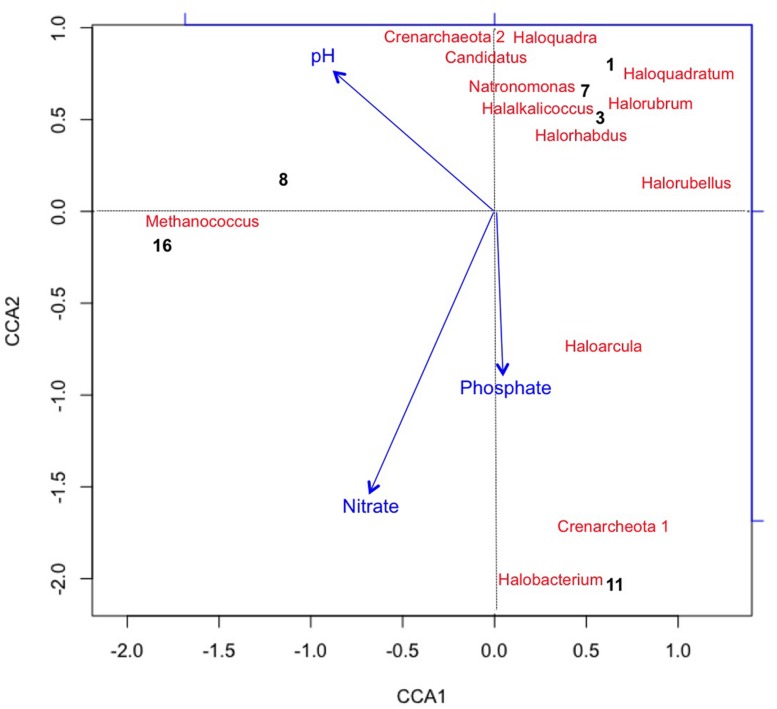
**Canonical Correspondence Analysis (CCA) plotting chemical variables that correlated with variation in Archaeal taxa (with >2% relative abundance) and sample points.** Archaea generally lacked long term survivability in the pond. CCA1 = 81.8% and CCA2 = 57.3%.

Using CCA, the most significant water chemistry variables correlated with fluctuations in archaeal taxa were plotted in **Figure 4.** Taxa and time points were correlated to pH, nitrate, and phosphate (vectors in **Figure 4**), and the temporal variation in archaeal taxa was observed as the pH increased on day 8 and 16 most likely as a consequence of photosynthesis. Not only does CCA provide insight into the environmental factors influencing the community structure, but it also suggests potential interactions occurring between taxa. The appearance and subsequent decline of plotted taxa was the most influential variable on discrepancies between the time points. For instance, day 8 is more similar to day 16 than other time points due to the loss of three archaeal taxa from day 7 to 8. As discussed, the low levels of salinity were an influential factor associated to the rapid decline of halophilic taxa.

**Figure 4** also shows that *Methanococcus maripaludis* was most associated with the variation in CCA1 as it was the only detectable archaeal taxa remaining at the last time point on day 16. *M. maripauldis* was detected on days 1, 3, 8, and 16, but not detected on days 7 and 11. While known Methanococci are strict anaerobes, there could be micro-anaerobic niches in the raceway pond related to biomass turnover. It is also possible that the sequences are detected at later time points due to PCR biases. Recent research has shown *M. maripauldis* can survive in anaerobic biofilms (Brileya et al., 2013); and therefore, it is possible that a small population was able to survive within a biofilm matrix on the walls of the raceway or paddle wheel. Its sporadic appearance may also be the result of collection methods that could have disturbed the biofilm or biofilm detachment. CCA2 was most influenced by nitrate and phosphate, which explained 38.4% of the variation in archaeal taxa.

**FIGURE 5:**
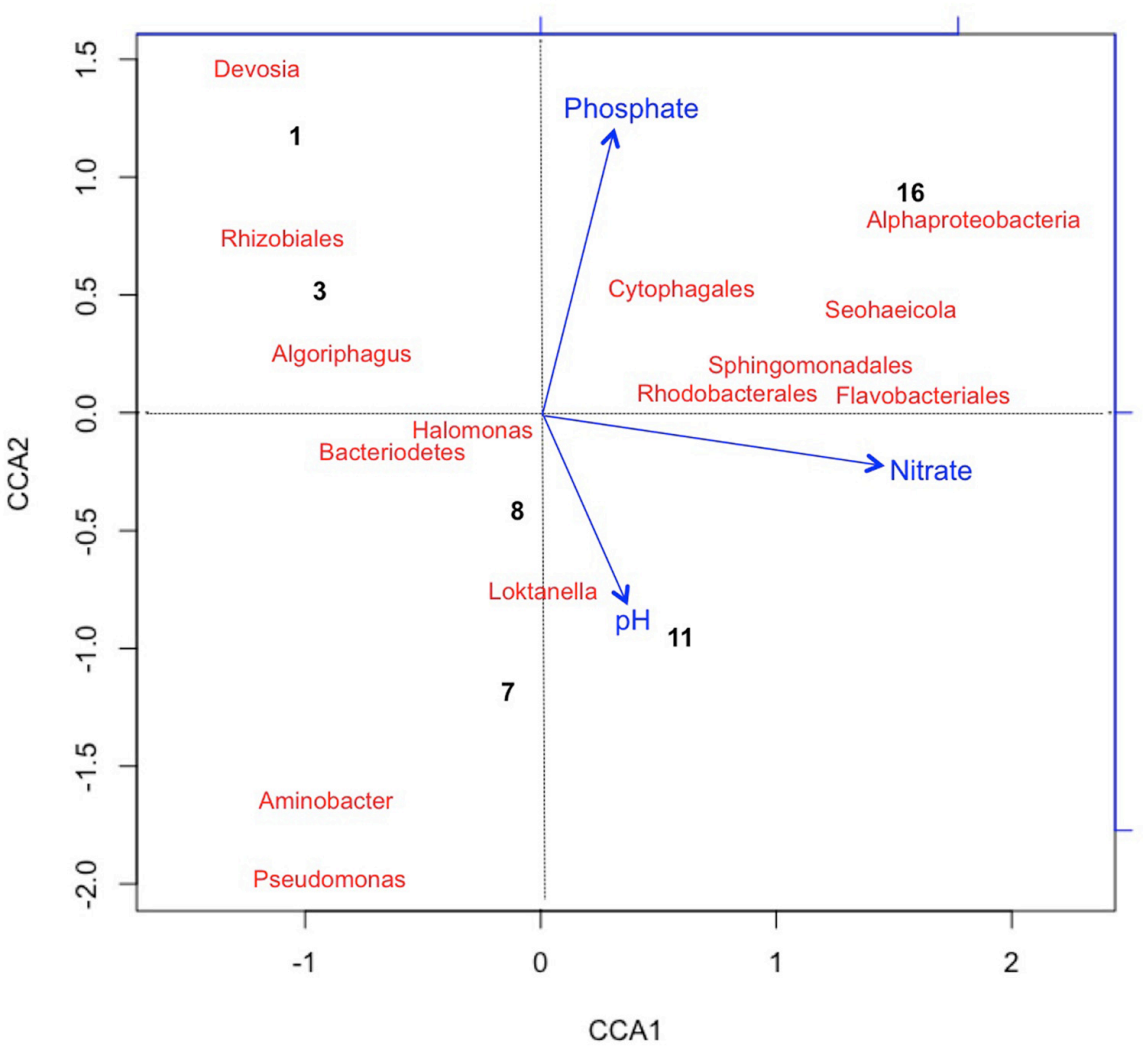
**Canonical Correspondence Analysis plotting chemical variables that correlated with variation in Bacterial taxa (with >2% relative abundance) and sample points.** Bacteria generally trended with an increase nitrate toward end of the experiment, possibly due to an increase in nitrifying taxa. *Pseudomonas* sp. was associated with the early time points (day 1 and 3). CCA1 = 60% and CCA2 = 8%.

We also applied the same CCA metrics for the bacterial taxa to visualize variance in taxa and time points as correlating with changes in chemical variables (**Figure 5**). CCA1 was predominately influenced by increased nitrate concentrations accounting for about 57.2% of the variance in bacterial taxa distribution. The first two time points were correlated with the initial halophilic bacterial taxa that were unable to maintain a population due to low salinity, aerobic conditions, and/or increasing pH (Post, 1977; Oren, 1994, 2006; Baxter et al., 2005; Tazi et al., 2014). The decrease in phosphate contributed approximately 30.5% of the observed variation in CCA2 and correlated with the increase in some of the most abundant taxa at day 16 [for example *Flavobacteriales* (*R*^2^ = 0.3) and *Proteobacteria* (*R*^2^ = 0.35)].

### Correlations Between Community Members

**FIGURE 6:**
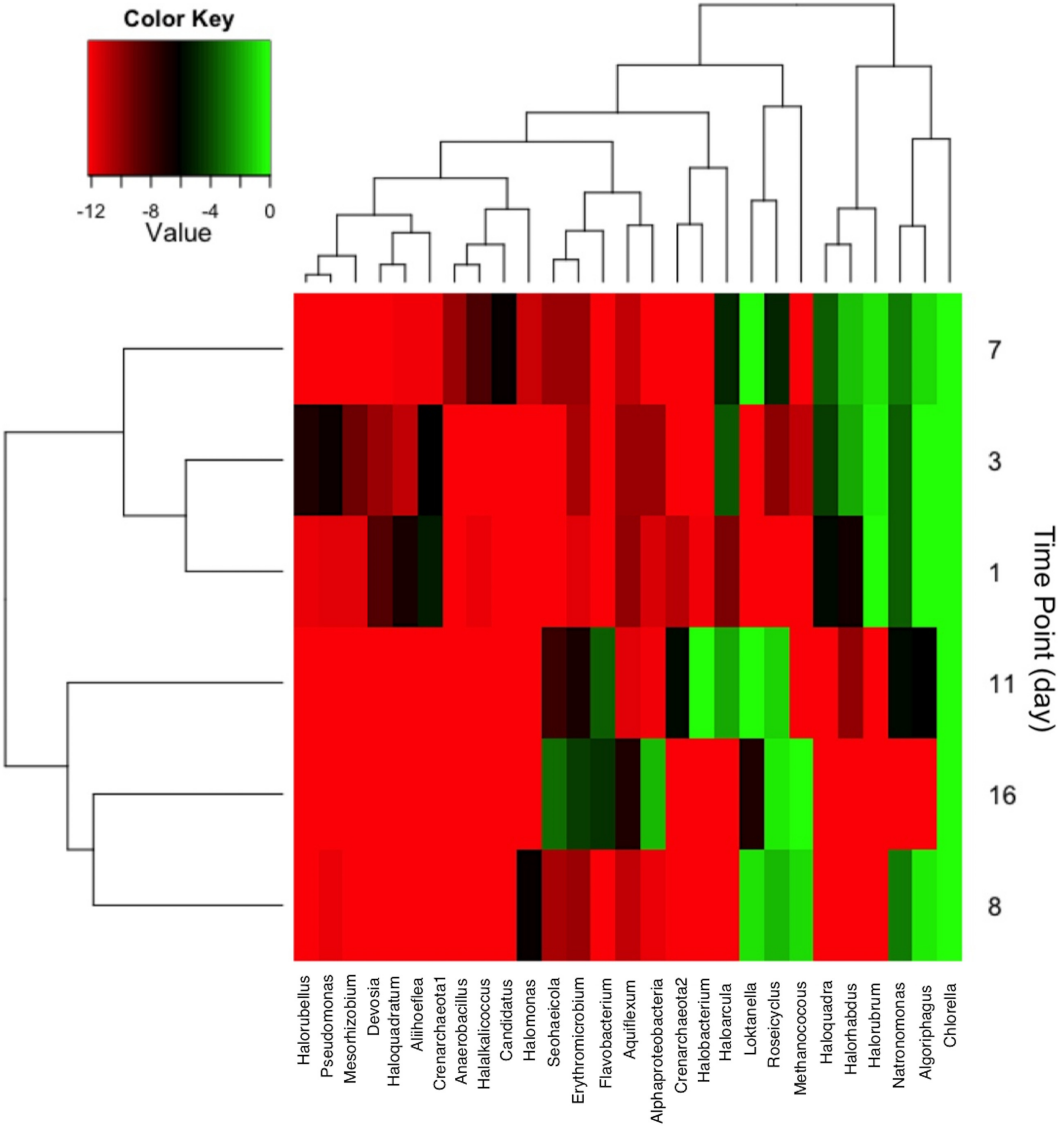
**Heat map showing taxa with greater than 2% relative abundance at each sampling time point.** Taxa co-occurrence and each time points were correlated and clustered according to relatedness as shown in the dendogram.

The distribution of community member occurrence is shown in **Figure 6**, illustrating the persistent *C. vulgaris* population. The upper dendogram clusters taxa by percent relative abundance and frequency of co-occurrence. As observed via CCA, the time points are grouped by declining archaeal sequences with the exception of Methanococcus at day 8 and 16. Post-transfer to 2,000 L, several bacterial OTUs clustered with day 11 and 16. *Flavobacterium* and *Erythromicrobium* are common groundwater/tap water organisms that were likely introduced during volume scale-up, but it is not known if the co-occurrence is direct or indirect. In addition, sequences indicative of *Loktanella* and *Roseicyclus* correlated with *Chlorella* on day 8, 11, and 16 during cultivation scale-up. Sequences indicative of *Algoriphagus* correlated with *Chlorella* during the 200 L cultivation but declined during the 2,000 L cultivation.

**FIGURE 7:**
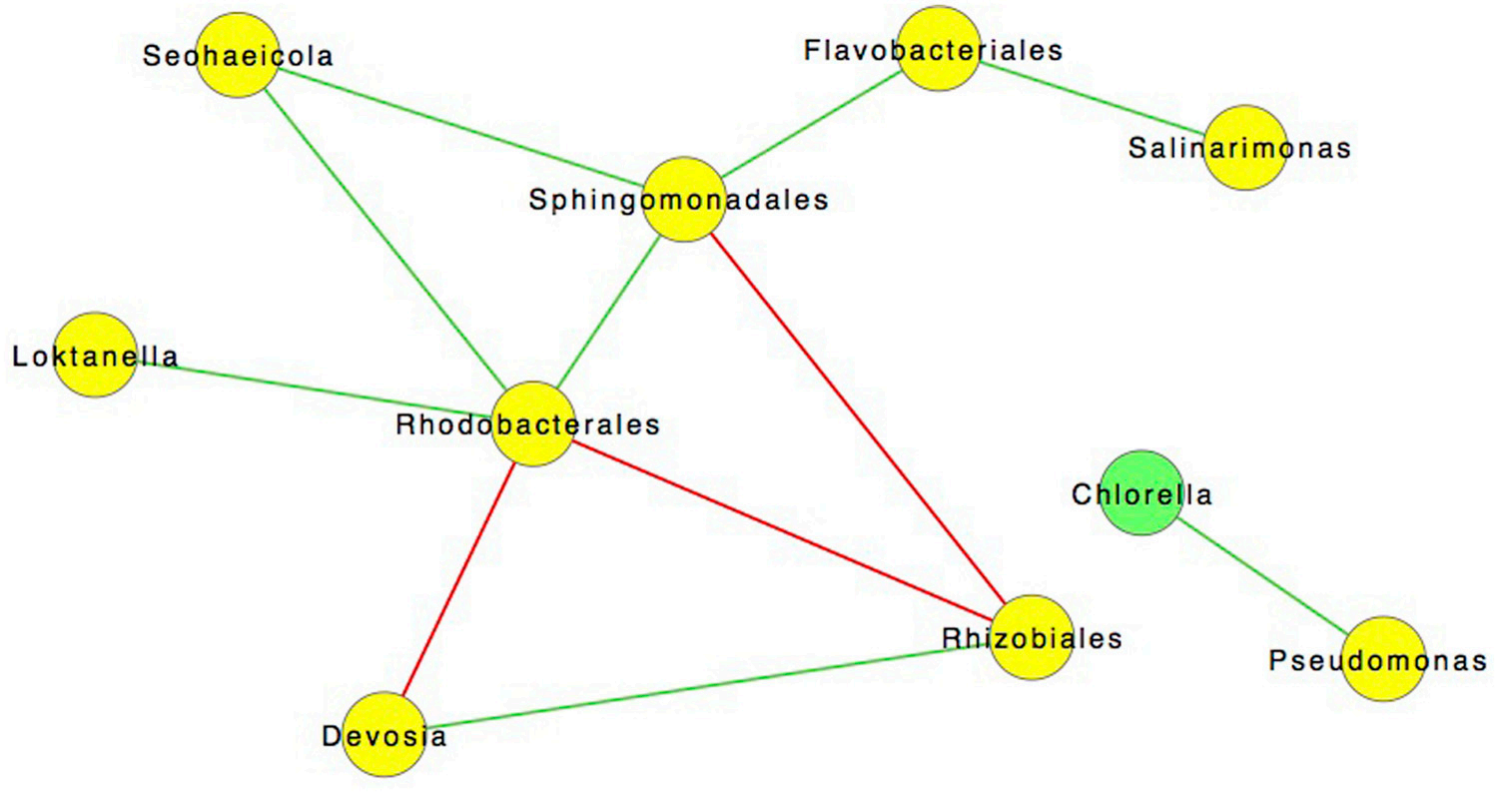
**SparCC network map showing significant (*p* < 0.05) of 122 Interactions with a 0.85 correlation between different OTUs incorporating all time points.** Green lines are indicative of positive interactions while red lines are negative.

However, co-occurrence does not infer a statistical correlation between taxa. In order to investigate possible correlations we used SparCC to construct community correlation networks. **Figure 7** shows a community network map of correlations between community members (excluding archaea due to their general absence after day 3) (Friedman and Alm, 2012). The most salient of these relationships is the positive 0.85 correlation between *C. vulgaris* and *Pseudomonas* sp. (*p* < 0.05). No other bacteria correlated with *C. vulgaris*, which was the predominant eukaryote. Previous studies have observed different species of *Pseudomonas* living in association with algae including *C. vulgaris* (Sapp et al., 2007). A symbiotic relationship between these organisms was described by Guo and Tong (2013) finding that *Pseudomonas sp*. fostered the growth of *C. vulgaris*. When in co-culture with *Pseudomonas* sp., the cell concentration of *C. vulgaris* was 1.4 times greater than that of axenic cultures under the same conditions. Scanning electron microscope (SEM) images revealed that the bacteria were living in the EPS or “phycosphere” of *C. vulgaris* (Guo and Tong, 2013).

The phycosphere, coined by Bell and Mitchell in 1972, is often colonized by bacteria (Bell and Mitchell, 1972; Sapp et al., 2007; Goecke et al., 2013). This specific niche facilitates a tight exchange of oxygen, carbon, and metabolites minimizing dilution (Sapp et al., 2008; Bruckner et al., 2011; Gärdes et al., 2012; Paul et al., 2012; Martin et al., 2014). Bacteria can provide the alga with sources of growth promoters (*e.g*., indole-3-acetic acid), and essential vitamins (e.g., cobalamin), while discouraging colonization by other potentially harmful microorganisms with antimicrobial metabolites (Gonzalez and Bashan, 2000; Croft et al., 2005, 2006). In return, bacteria have immediate access to algal exudates that can be a key source of fixed carbon (Bell and Mitchell, 1972; Sapp et al., 2007; Goecke et al., 2013). The correlation of *Pseudomonas* populations with *C. vulgaris* throughout the course of the pond experiment, even following the transfer to the larger raceway, may have contributed to the predominance of the algal culture under open conditions (**Figure 7**). The results suggest that symbiotic-associations could have relevant industrial applications that could result in increased biomass yields (Imase et al., 2008; Natrah et al., 2013). Further work is needed to discern the mechanism(s) of interactions that impact algal biomass and/or lipid accumulation in addition to confirmation of a direct and/or indirect relationship between these two organisms under the tested growth conditions.

## Conclusion

Our work demonstrated that the cultivation of a single algal strain in an open pond without the addition of antibiotics or herbicides can be successful. The use of high pH systems and alkaline adapted algal taxa could be a successful strategy for overcoming some of the constraints associated with large-scale biomass production in open systems. Furthermore, certain phycosphere associations could enhance biomass yields and deter colonization by detrimental populations. Further work is needed to determine the longevity and stability of open, outdoor cultivation systems for the production of algal biomass and/or biomolecules.

## Conflict of Interest Statement

The authors declare that the research was conducted in the absence of any commercial or financial relationships that could be construed as a potential conflict of interest.
